# *N*,*N*′-(1,4-Phenyl­ene)bis­(2-bromo-2-methyl­propanamide)

**DOI:** 10.1107/S1600536812003479

**Published:** 2012-02-24

**Authors:** N. Haridharan, V. Ramkumar, R. Dhamodharan

**Affiliations:** a Department of Chemistry, IIT Madras, Chennai, Tamil nadu, India

## Abstract

The mol­ecular structure of the title compound, C_14_H_18_Br_2_N_2_O_2_, has one half-mol­ecule in the asymmetric unit. The mol­ecule has a crystallographic inversion centre in the middle of the benzene ring. The C—C—N—C torsion angle between the benzene ring and the bromo­amide group is 149.2 (7)°. The crystal is stabilized by a strong inter­molecular N—H⋯O bond and weak C—H⋯O inter­actions. These contacts give rise to a three-dimensional network.

## Related literature

For the use of the title compound as an initiator in atom transfer radical polymerization and other polymerization studies, see: Ashraf *et al.* (1994 ▶); Domenicano *et al.* (1977 ▶); Kuipers *et al.* (1989 ▶); Matyjaszewski & Xia (2001 ▶); Miroshnikova *et al.* (2007 ▶); Rollison *et al.* (2006 ▶). For similar structures, see: Haridharan *et al.* (2010 ▶).
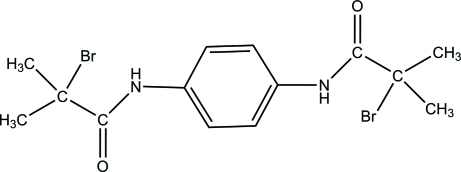


## Experimental

### 

#### Crystal data

C_14_H_18_Br_2_N_2_O_2_
*M* *_r_* = 406.12
Monoclinic, 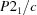

*a* = 13.834 (3) Å
*b* = 6.4746 (13) Å
*c* = 9.4642 (18) Å
β = 103.807 (9)°
*V* = 823.2 (3) Å^3^
*Z* = 2
Mo *K*α radiation
μ = 4.93 mm^−1^
*T* = 298 K
0.35 × 0.22 × 0.05 mm

#### Data collection

Bruker APEXII CCD area-detector diffractometer
Absorption correction: multi-scan (*SADABS*; Bruker, 2004 ▶) *T*_min_ = 0.278, *T*_max_ = 0.791
1834 measured reflections
1834 independent reflections
664 reflections with *I* > 2σ(*I*)
*R*_int_ = 0.000

#### Refinement

*R*[*F* ^2^ > 2σ(*F* ^2^)] = 0.056
*wR*(*F* ^2^) = 0.146
*S* = 0.94
1834 reflections
93 parameters
H-atom parameters constrained
Δρ_max_ = 0.54 e Å^−3^
Δρ_min_ = −0.56 e Å^−3^

### 

Data collection: *APEX2* (Bruker, 2004 ▶); cell refinement: *APEX2* and *SAINT-Plus* (Bruker, 2004 ▶); data reduction: *SAINT-Plus* and *XPREP* (Bruker, 2004 ▶); program(s) used to solve structure: *SHELXS97* (Sheldrick, 2008 ▶); program(s) used to refine structure: *SHELXL97* (Sheldrick, 2008 ▶); molecular graphics: *ORTEP-3* (Farrugia, 1997 ▶); software used to prepare material for publication: *SHELXL97*.

## Supplementary Material

Crystal structure: contains datablock(s) global, I. DOI: 10.1107/S1600536812003479/bv2195sup1.cif

Structure factors: contains datablock(s) I. DOI: 10.1107/S1600536812003479/bv2195Isup2.hkl

Supplementary material file. DOI: 10.1107/S1600536812003479/bv2195Isup3.cml

Additional supplementary materials:  crystallographic information; 3D view; checkCIF report

## Figures and Tables

**Table 1:** Hydrogen-bond geometry (Å, °)

*D*—H⋯*A*	*D*—H	H⋯*A*	*D*⋯*A*	*D*—H⋯*A*
N1—H1*A*⋯O1^i^	0.86	2.23	3.057 (7)	162
C7—H7*B*⋯O1^i^	0.96	2.57	3.503 (9)	164

Symmetry code: (i) 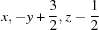.

**Fig. 1.**
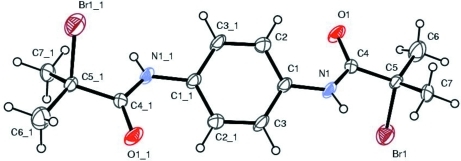
ORTEP of the molecule with atoms represented as 30% probability ellipsoids.

## supplementary crystallographic information

### Comment

The title compound C_14_ H_18_ Br_2_ N_2_ O_2_ is a difunctional aromatic amide based derivative, which is used as an initiator in Atom Transfer Radical Polymerization (ATRP) (Matyjaszewski *et al.*, (2001). We have already reported a similar ATRP initiator (Haridharan *et al.*, 2010). The title compound reported here is a similar derivative with a diamide functionality (Domenicano *et al.* (1977); Kuipers *et al.* (1989). It is mainly used as a component of engineering polymers and composites (Ashraf *et al.*, 1994). It is also an ingredient in hair dyes.

*p*-Phenylenediamine is a precursor to aramid plastics and fibers such as Kevlar (Rollison *et al.*, 2006). *p*-Phenylenediamine is also used as a developing agent in the color photographic film development process, reacting with the silver grains in the film and creating the colored dyes that form the image. *p*-Phenylenediamine derivatives such as chloroquine are the most important and widely used class of drugs for treatment of malaria (Miroshnikova *et al.*, 2007). In the title compound C_14_H_18_Br_2_N_2_O_2_, the torsion angle between the phenyl ring and the bromo amide group is 149.2 (7)° (C3—C1—N1—C4). The molecule has a crystallographic inversion centre in the middle of the phenyl ring. The crystal is stabilized by a strong intermolecular N—H···O bonding and weak C-H···O interactions. These contacts gives rise to a three dimensional network.

### Experimental

*p*-Phenylene diamine (5 g, 0.012 moles), triethylamine (12 g, 0.05 moles) and THF (400 ml) were placed in a 3-neck round bottomed flask. Bromoisobutyrl bromide (13.7 g, 0.05 moles) was added slowly, using a syringe, with stirring, upon which a brown precipitate of triethylammonium bromide was formed. The mixture was left to react for 12 h, with stirring. Subsequently, triethylammonium bromide, the precipitate was removed by filtration and the THF was removed by rotary evaporation. The resulting crude product was dissolved in ethyl acetate, washed with bicarbonate solution and then with water thrice followed by brine solution and dried over anhydrous sodium sulfate. The resulting solvent was removed by rotary evaporation. The product was purified by column chromatography technique using 10% ethyl acetate in hexane as the eluent to obtain pure initiator as a bright yellow solid. Recrystallization of the compound from hexane gave X-ray diffraction quality crystals of N,N'-(1,4-phenylene)bis(2-bromo-2-methylpropanamide).

### Refinement

All hydrogen atoms were fixed geometrically and allowed to ride on the parent carbon atoms, with aromatic C—H = 0.93 Å, methyl C—H = 0.96 Å and methylene C—H = 0.97 Å. The displacement parameters were set for phenyl and methylene H atoms at *U*_iso_(H) = 1.2*U*_eq_(C) and methyl H atoms at *U*_iso_(H) = 1.5*U*_eq_(C).

The crystal data was collected up to 0.73 Å resolution, the crystal (the largest available) still diffracted quite weakly at high angle. On repeated crystallization we could get only small crystals which weakly diffracted. The data completeness we could get was only 93.6%.

### Crystal data

**Table:**

C_14_H_18_Br_2_N_2_O_2_	*F*(000) = 404
*M**_r_* = 406.12	*D*_x_ = 1.638 Mg m^−^^3^
Monoclinic, *P*2_1_/*c*	Mo *K*α radiation, λ = 0.71073 Å
Hall symbol: -P 2ybc	Cell parameters from 1219 reflections
*a* = 13.834 (3) Å	θ = 0.0–0.0°
*b* = 6.4746 (13) Å	µ = 4.93 mm^−^^1^
*c* = 9.4642 (18) Å	*T* = 298 K
β = 103.807 (9)°	Block, colourless
*V* = 823.2 (3) Å^3^	0.35 × 0.22 × 0.05 mm
*Z* = 2	

### Data collection

**Table:**

Bruker APEXII CCD area-detector diffractometer	1834 independent reflections
Radiation source: fine-focus sealed tube	664 reflections with *I* > 2σ(*I*)
Graphite monochromator	*R*_int_ = 0.000
φ and ω scans	θ_max_ = 27.8°, θ_min_ = 3.0°
Absorption correction: multi-scan (*SADABS*; Bruker, 2004)	*h* = −18→17
*T*_min_ = 0.278, *T*_max_ = 0.791	*k* = 0→8
1834 measured reflections	*l* = 0→11

### Refinement

**Table:**

Refinement on *F*^2^	Primary atom site location: structure-invariant direct methods
Least-squares matrix: full	Secondary atom site location: difference Fourier map
*R*[*F*^2^ > 2σ(*F*^2^)] = 0.056	Hydrogen site location: inferred from neighbouring sites
*wR*(*F*^2^) = 0.146	H-atom parameters constrained
*S* = 0.94	*w* = 1/[σ^2^(*F*_o_^2^) + (0.0575*P*)^2^] where *P* = (*F*_o_^2^ + 2*F*_c_^2^)/3
1834 reflections	(Δ/σ)_max_ < 0.001
93 parameters	Δρ_max_ = 0.54 e Å^−^^3^
0 restraints	Δρ_min_ = −0.56 e Å^−^^3^

### Special details

**Table:**

Geometry. All esds (except the esd in the dihedral angle between two l.s. planes) are estimated using the full covariance matrix. The cell esds are taken into account individually in the estimation of esds in distances, angles and torsion angles; correlations between esds in cell parameters are only used when they are defined by crystal symmetry. An approximate (isotropic) treatment of cell esds is used for estimating esds involving l.s. planes.
Refinement. Refinement of F^2^ against ALL reflections. The weighted R-factor wR and goodness of fit S are based on F^2^, conventional R-factors R are based on F, with F set to zero for negative F^2^. The threshold expression of F^2^ > 2sigma(F^2^) is used only for calculating R-factors(gt) etc. and is not relevant to the choice of reflections for refinement. R-factors based on F^2^ are statistically about twice as large as those based on F, and R- factors based on ALL data will be even larger.

### Fractional atomic coordinates and isotropic or equivalent isotropic displacement parameters (Å^2^)

**Table:**

	*x*	*y*	*z*	*U*_iso_*/*U*_eq_	
Br1	0.90343 (6)	0.80012 (14)	0.54184 (10)	0.0699 (4)	
O1	0.7011 (3)	0.5876 (8)	0.6804 (5)	0.0520 (14)	
N1	0.6647 (4)	0.7640 (8)	0.4699 (6)	0.0436 (16)	
H1A	0.6809	0.7779	0.3882	0.052*	
C1	0.5804 (5)	0.8774 (11)	0.4872 (7)	0.0364 (18)	
C2	0.5188 (5)	0.8134 (11)	0.5735 (8)	0.050 (2)	
H2	0.5308	0.6887	0.6234	0.060*	
C3	0.5607 (5)	1.0646 (13)	0.4144 (7)	0.045 (2)	
H3	0.6021	1.1089	0.3561	0.054*	
C4	0.7212 (5)	0.6389 (10)	0.5664 (8)	0.0366 (18)	
C5	0.8137 (4)	0.5549 (10)	0.5272 (7)	0.0387 (18)	
C6	0.8682 (6)	0.4020 (14)	0.6398 (9)	0.079 (3)	
H6A	0.8881	0.4692	0.7327	0.118*	
H6B	0.9261	0.3518	0.6116	0.118*	
H6C	0.8250	0.2884	0.6465	0.118*	
C7	0.7975 (5)	0.4668 (11)	0.3732 (7)	0.051 (2)	
H7A	0.8601	0.4226	0.3567	0.076*	
H7B	0.7693	0.5714	0.3036	0.076*	
H7C	0.7529	0.3512	0.3627	0.076*	

### Atomic displacement parameters (Å^2^)

**Table:**

	*U*^11^	*U*^22^	*U*^33^	*U*^12^	*U*^13^	*U*^23^
Br1	0.0573 (6)	0.0714 (7)	0.0882 (8)	−0.0096 (5)	0.0316 (4)	−0.0175 (5)
O1	0.056 (3)	0.074 (4)	0.035 (3)	0.008 (3)	0.028 (3)	0.009 (3)
N1	0.053 (4)	0.056 (4)	0.032 (4)	0.018 (3)	0.028 (3)	0.011 (3)
C1	0.040 (4)	0.046 (5)	0.028 (4)	0.003 (3)	0.018 (3)	0.000 (4)
C2	0.056 (5)	0.050 (5)	0.052 (5)	0.013 (4)	0.027 (4)	0.018 (4)
C3	0.043 (4)	0.066 (6)	0.033 (5)	0.010 (4)	0.026 (3)	0.009 (4)
C4	0.045 (4)	0.032 (5)	0.038 (5)	0.007 (3)	0.021 (4)	0.004 (4)
C5	0.037 (4)	0.044 (5)	0.041 (5)	0.012 (4)	0.022 (3)	0.012 (4)
C6	0.095 (7)	0.092 (7)	0.062 (6)	0.042 (6)	0.044 (5)	0.028 (5)
C7	0.076 (5)	0.041 (5)	0.046 (5)	0.013 (4)	0.036 (4)	−0.006 (4)

### Geometric parameters (Å, º)

**Table:**

Br1—C5	2.000 (7)	C3—H3	0.9300
O1—C4	1.223 (7)	C4—C5	1.516 (8)
N1—C4	1.326 (8)	C5—C6	1.517 (9)
N1—C1	1.421 (8)	C5—C7	1.531 (9)
N1—H1A	0.8600	C6—H6A	0.9600
C1—C2	1.377 (8)	C6—H6B	0.9600
C1—C3	1.389 (9)	C6—H6C	0.9600
C2—C3^i^	1.381 (9)	C7—H7A	0.9600
C2—H2	0.9300	C7—H7B	0.9600
C3—C2^i^	1.381 (9)	C7—H7C	0.9600
			
C4—N1—C1	127.0 (5)	C6—C5—C7	111.2 (6)
C4—N1—H1A	116.5	C4—C5—Br1	104.2 (4)
C1—N1—H1A	116.5	C6—C5—Br1	105.8 (5)
C2—C1—C3	118.8 (6)	C7—C5—Br1	108.0 (4)
C2—C1—N1	123.6 (7)	C5—C6—H6A	109.5
C3—C1—N1	117.6 (5)	C5—C6—H6B	109.5
C1—C2—C3^i^	119.7 (7)	H6A—C6—H6B	109.5
C1—C2—H2	120.2	C5—C6—H6C	109.5
C3^i^—C2—H2	120.2	H6A—C6—H6C	109.5
C2^i^—C3—C1	121.6 (6)	H6B—C6—H6C	109.5
C2^i^—C3—H3	119.2	C5—C7—H7A	109.5
C1—C3—H3	119.2	C5—C7—H7B	109.5
O1—C4—N1	123.5 (6)	H7A—C7—H7B	109.5
O1—C4—C5	120.1 (6)	C5—C7—H7C	109.5
N1—C4—C5	116.4 (6)	H7A—C7—H7C	109.5
C4—C5—C6	111.7 (5)	H7B—C7—H7C	109.5
C4—C5—C7	115.2 (6)		
			
C4—N1—C1—C2	−29.5 (11)	C1—N1—C4—C5	−173.4 (6)
C4—N1—C1—C3	149.4 (7)	O1—C4—C5—C6	2.8 (10)
C3—C1—C2—C3^i^	0.1 (12)	N1—C4—C5—C6	−175.8 (7)
N1—C1—C2—C3^i^	179.0 (6)	O1—C4—C5—C7	130.9 (7)
C2—C1—C3—C2^i^	−0.2 (12)	N1—C4—C5—C7	−47.8 (8)
N1—C1—C3—C2^i^	−179.1 (6)	O1—C4—C5—Br1	−111.0 (6)
C1—N1—C4—O1	8.0 (11)	N1—C4—C5—Br1	70.4 (7)

Symmetry code: (i) −*x*+1, −*y*+2, −*z*+1.

### Hydrogen-bond geometry (Å, º)

**Table:**

*D*—H···*A*	*D*—H	H···*A*	*D*···*A*	*D*—H···*A*
N1—H1*A*···O1^ii^	0.86	2.23	3.057 (7)	162
C7—H7*B*···O1^ii^	0.96	2.57	3.503 (9)	164

Symmetry code: (ii) *x*, −*y*+3/2, *z*−1/2.
